# Supplementary material for: Investigation of clinical characteristics and genome associations in the ‘UK Lipoedema’ cohort
Source: PLoS One. 2022 Oct 13;17(10):e0274867. doi: 10.1371/journal.pone.0274867 (PMC9560129; doi:10.1371/journal.pone.0274867)
Supplement: S1 File — (PDF) [file pone.0274867.s001.pdf]

Investigation of clinical characteristics and genome associations in the 'UK Lipoedema' cohort

Grigoriadis *et al.*

Supplementary Methods

Case ascertainment

The diagnosis of lipoedema and recruitment to the study used the following inclusion and exclusion criteria based on consensus opinions of clinical experts.

List of inclusion criteria (as summarized in Table 1)

- Female.
- Age of onset (ideally below 35 years).
- BMI ≤40 kg/m^2.
- Waist-hip ratio (WHR) ≤0.85*.
- No or minimal central (android) obesity.
- Bilateral and symmetrical fat hypertrophy on lower limbs (e.g. "saddlebags" fat distribution on hips or steatopygia, medial knee "fat pads"), usually soft and "doughy"/"floppy" to the touch.
- Spared feet.
- Persistent enlargement (with no significant effect from overnight elevation)
- White British ethnicity (only applicable for the GWAS).

*The WHO advises that a healthy WHR is ≤0.85 for women [2], WHR >0.85 indicates abdominal obesity.

Individuals were excluded from the study if any of the following criteria were identified.

List of exclusion criteria

- Lymphoedema skin changes (hyperkeratosis or papillomatosis).
- Firm, fibrotic swelling suggesting chronic lymphoedema.
- History of episodes of acute cellulitis.
- No significant comorbidities (e.g. diabetes or renal disease).
- Morbid obesity

Each patient was carefully assessed through clinical examination and face-to-face interview and a general detailed medical history was taken and data collected on:

- weight, height, BMI
- waist and hip circumference, WHR
- family history of lipoedema
- arm swelling
- hypermobility
- age of onset of lipoedema
- age of puberty
- limb volume response to dieting
- easy bruising of affected areas
- tenderness or pain (self-reported, information obtained through interview)
- history or presence of venous disease (self-reported and inspected)
- history or presence of orthostatic oedema (self-reported and inspected)

Clinical summaries, including medical photographs obtained with patient consent, were reviewed by at least two specialists, and all available medical case files studied. Patients whose BMI was above 40 kg/m^2 or WHR above 0.85 at the time of the recruitment consultation were included in the study, but only if they had been known to the senior clinician for several years and historically would have fallen within the inclusion criteria. If the patient was new to our service and did not fulfil the major inclusion criteria, they were not included in the analyses.

The waist was measured in centimetres at the narrowest part of the abdomen usually close to the umbilicus. The hips were measured (in cm) by locating the greater trochanter of the femur and measuring at that level, usually the widest portion of the buttocks. Waist-hip ratio (WHR) was then calculated as the waist circumference divided by hip circumference.

Some of the data collected need to be interpreted with caution as they included subjective measures based on self-reporting, such as easy bruising, tenderness/pain and response to diet. For example, the responsiveness to diet was assessed through the question *"If responsive to dieting and there is weight loss; is it disproportionate with less weight lost from legs (and arms) than the rest of the body?".* However, the perception of what dieting and weight-loss entails can be subjective, and we had no means of following up on this.

Age of onset of lipoedema is self-reported in most cases. Disease duration was calculated by subtracting the age of onset from age at recruitment date.

Hypermobility or joint laxity was checked at elbows, knees, small joints of the hands and back (Beighton score). If hypermobile in four or more joints, the recruit was scored as hypermobile.

Venous problems were assessed through clinical assessment and categorized into 'mild superficial' (equivalent to CEAP C1) and 'uncomplicated varicose veins' (equivalent to CEAP C2). A few cases self-reported having undergone surgery for varicose veins (classed as CEAP C3). However, chronic venous disease was not formerly assessed with e.g. duplex scan. Regarding the assessment of orthostatic

oedema, we did not differentiate between lymphoedema or chronic oedema due to e.g. venous hypertension.

It was also recorded if the patient had undergone liposuction or bariatric surgery. Only cases known to us with clinically confirmed lipoedema prior to these interventions were included in the study.

Supplementary References

1. Centre for Public Health Excellence at N, National Collaborating Centre for Primary C. National Institute for Health and Clinical Excellence: Guidance. Obesity: The Prevention, Identification, Assessment and Management of Overweight and Obesity in Adults and Children. London: National Institute for Health and Clinical Excellence (UK)

2. WHO. Waist circumference and waist-hip ratio : report of a WHO expert consultation, Geneva, 8-11 December 2008: World Health Organization; 2011.

3. Torrance N, Smith BH, Lee AJ, Aucott L, Cardy A, Bennett MI. Analysing the SF-36 in population-based research. A comparison of methods of statistical approaches using chronic pain as an example. J Eval Clin Pract. 2009;15(2):328-34. Epub 2009/04/02. doi: 10.1111/j.1365-2753.2008.01006.x. PubMed PMID: 19335493.

4. Sahle B, Slewa-Younan S, Melaku Y, Ling L, Renzaho A. A Bi-Directional Association Between Weight Change and Health-Related Quality of Life: Evidence From the 11-year Follow-Up of 9916 Community-Dwelling Adults. Quality of life research : an international journal of quality of life aspects of treatment, care and rehabilitation. 2020;29(6). doi: 10.1007/s11136-020-02423-7. PubMed PMID: 31938964.

5. Bowling A, Bond M, Jenkinson C, Lamping D. Short Form 36 (SF-36) Health Survey questionnaire: which normative data should be used? Comparisons between the norms provided by the Omnibus Survey in Britain, the Health Survey for England and the Oxford Healthy Life Survey. Journal of Public Health. 1999;21(3):255-70. doi: 10.1093/pubmed/21.3.255.
