# Supplementary figures and images for: Investigation of clinical characteristics and genome associations in the ‘UK Lipoedema’ cohort
Source: PLoS One. 2022 Oct 13;17(10):e0274867. doi: 10.1371/journal.pone.0274867 (PMC9560129; doi:10.1371/journal.pone.0274867)

**S1 Fig. Distribution of BMI, WHR and waist circumference.**

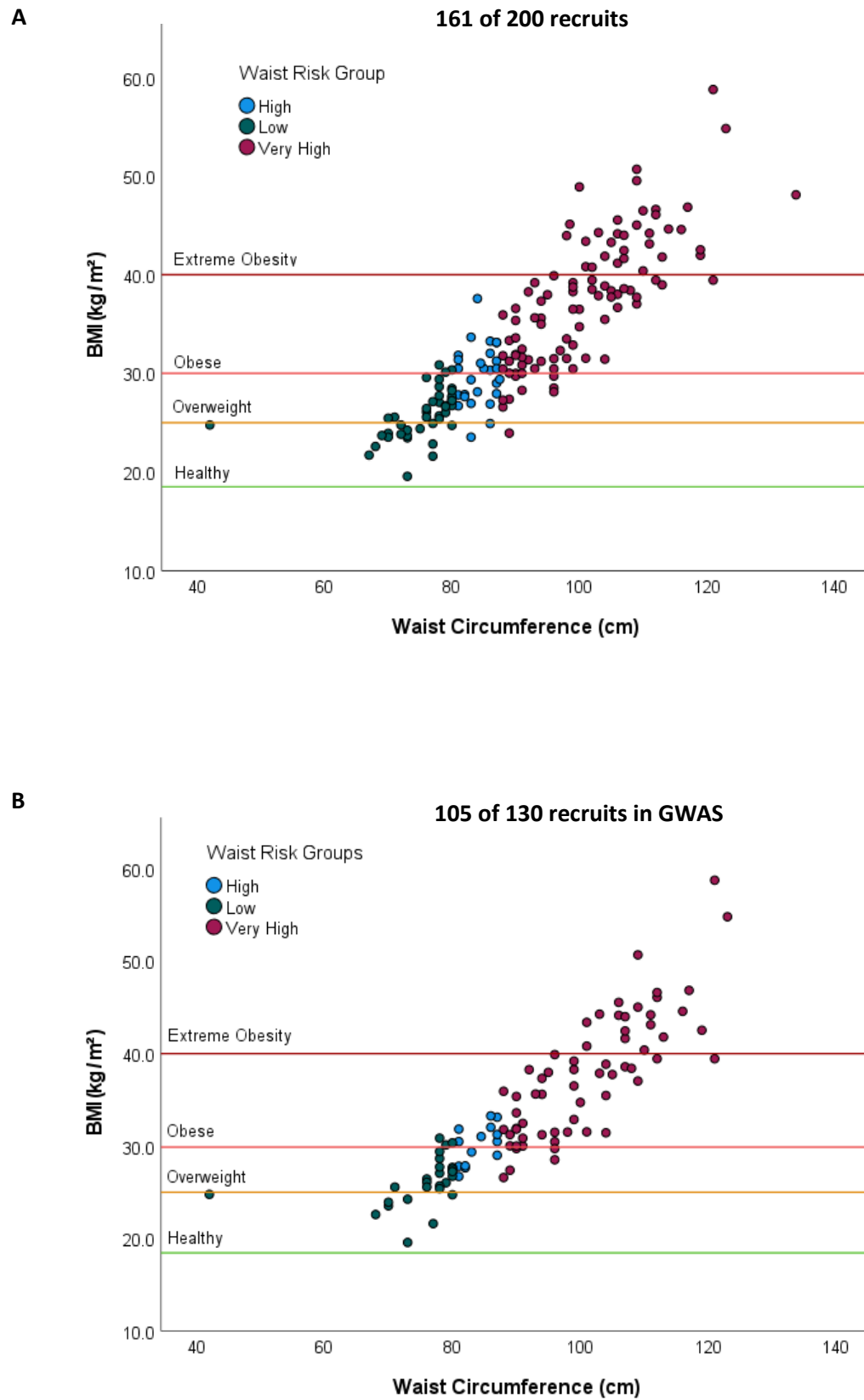

**C**

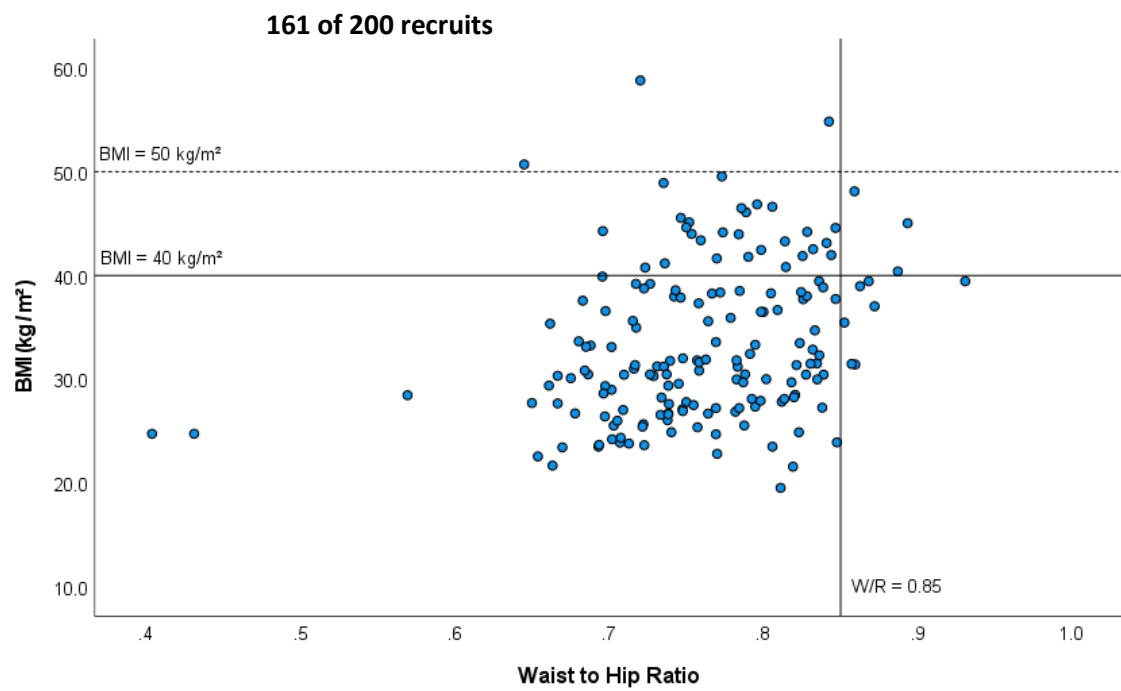

**D**

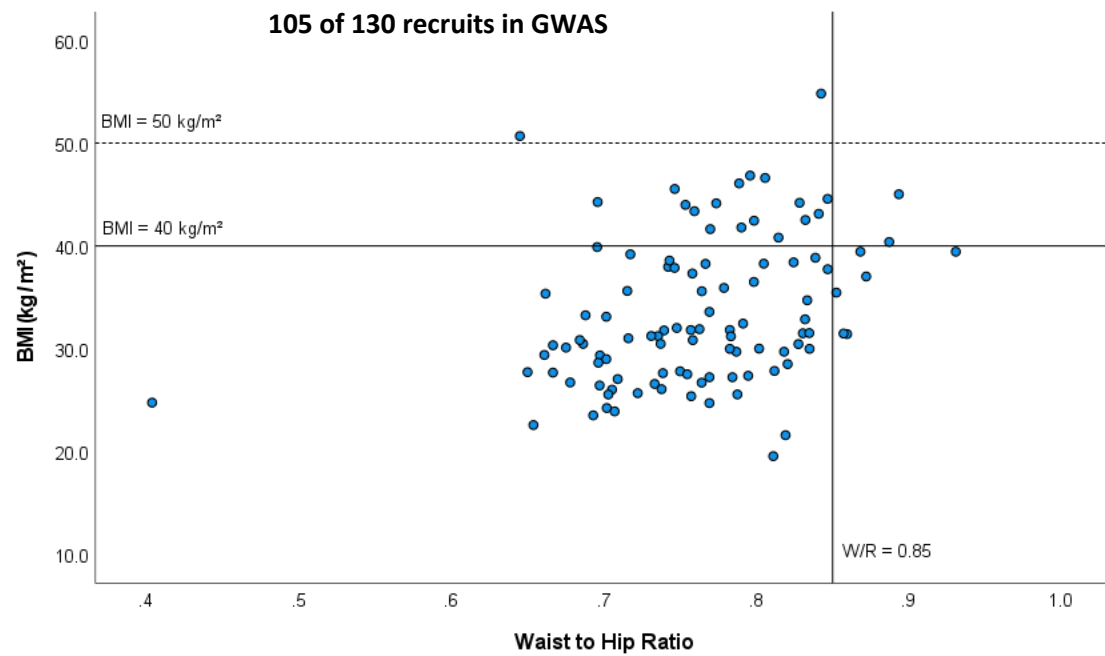

Supplement: S1 Fig — Of the 200 lipoedema cases recruited to the ‘UK Lipoedema’ cohort, we have anthropometric data for 161 (A and C). 130 individuals of white British descent were selected for GWAS of which 105 have been plotted in (B and D). (A, B) Waist circumference vs BMI show that many individuals fall in the overweight (BMI over 25 kg/m2; yellow line) and obese (BMI over 30 kg/m2; red line) categories. According to the NHS waist measurement guidelines for white European women, a waistline < 80cm is low risk (in green), high risk 80–88cm (in blue) and very high risk > 88cm (in red) of developing diseases such as type 2 diabetes, hypertension, coronary heart disease, cancer and stroke [1]. (C, D) Waist-hip ratio (WHR) vs BMI show that the majority of cases included in the study have a WHR < 0.85, which according to WHO guidelines is healthy [2]. Any cases outside the region of inclusion, i.e. the cases with a WHR > 0.85 (and BMI > 40), have been carefully investigated by the clinicians involved before being included in the study (see Supplementary Methods, S1 File, for details on case ascertainment). (PDF) [file pone.0274867.s010.pdf]

**S2 Fig.** Plot of the SNPs showing evidence of colocalization from LocusFocus.

**A.**

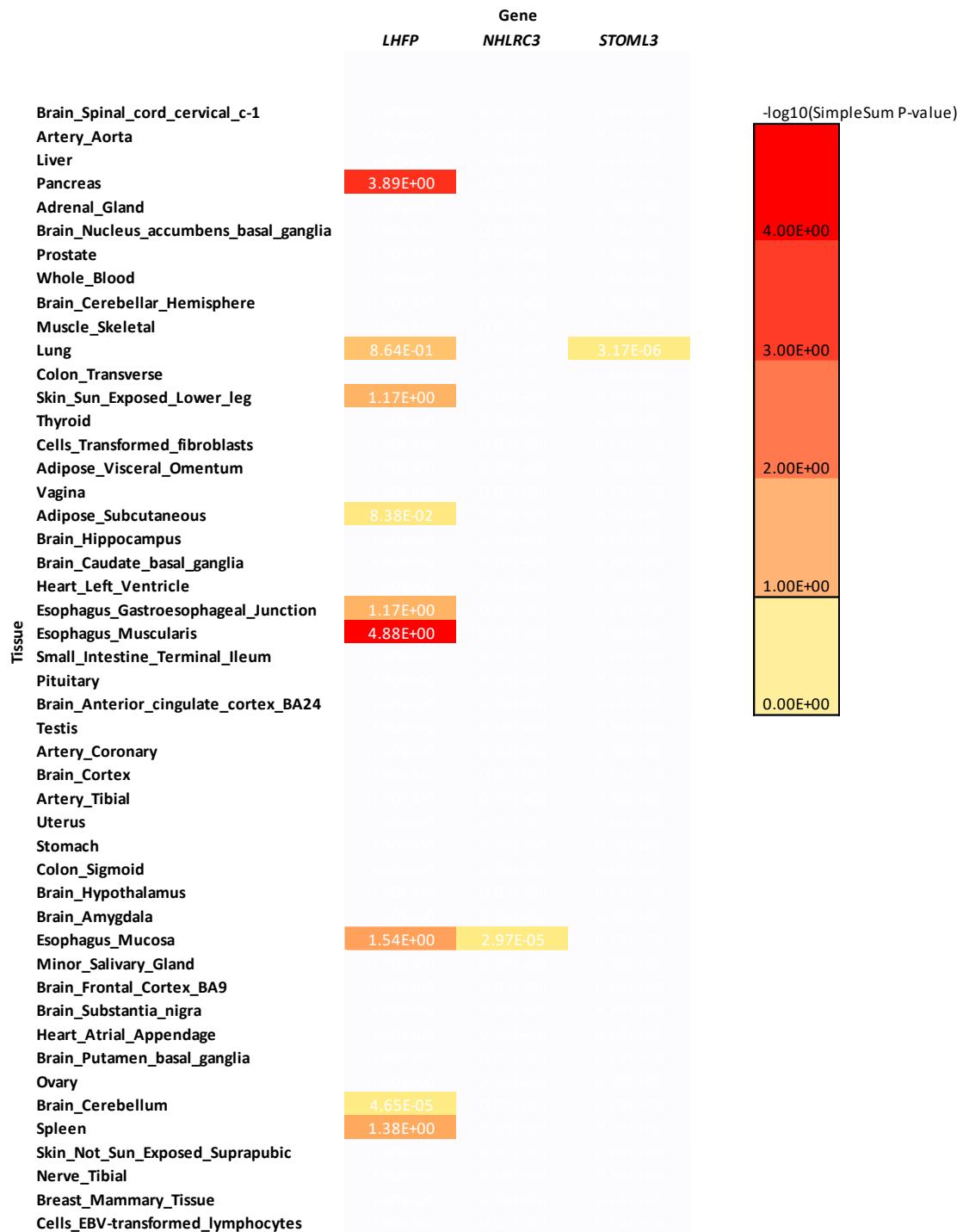

**B.**

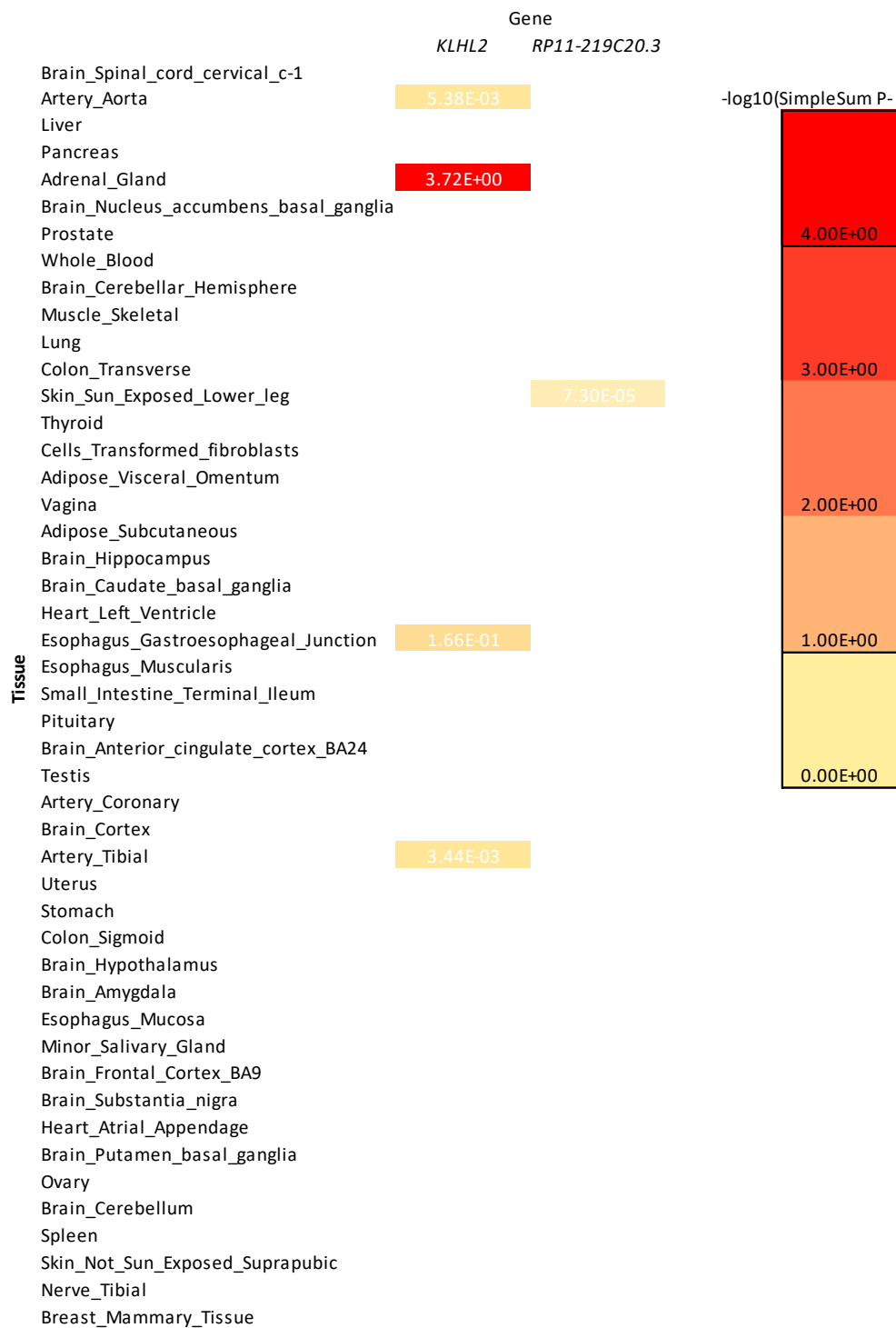

Supplement: S2 Fig — Heatmap shown summarize the SS colocalization tests for all the genes in the ±500 kb region of each SNP across all 54 GTEx tissues. Strength of colocalization is coloured from yellow (low -log10(P)) to red (high -log10(P)). White indicates either no eQTL data or gene-tissue pair does not have significant eQTL signal after Bonferroni correction. A. Heatmap shows results where SSP> 0 for genes in the region around rs1409440 illustrating the colocalization of LHFPL6 eQTLs across multiple tissues. B. Heatmap shows results where SSP>0 for genes in the region around rs9308098 illustrating colocalization of the KLHL2 eQTL in adrenal tissue. (PDF) [file pone.0274867.s011.pdf]
