# Supplementary material for: Investigation of clinical characteristics and genome associations in the ‘UK Lipoedema’ cohort
Source: PLoS One. 2022 Oct 13;17(10):e0274867. doi: 10.1371/journal.pone.0274867 (PMC9560129; doi:10.1371/journal.pone.0274867)

S3 Fig. Radar diagram showing the mean score for each of the 8 domains from the SF-36 Quality of Life questionnaire.

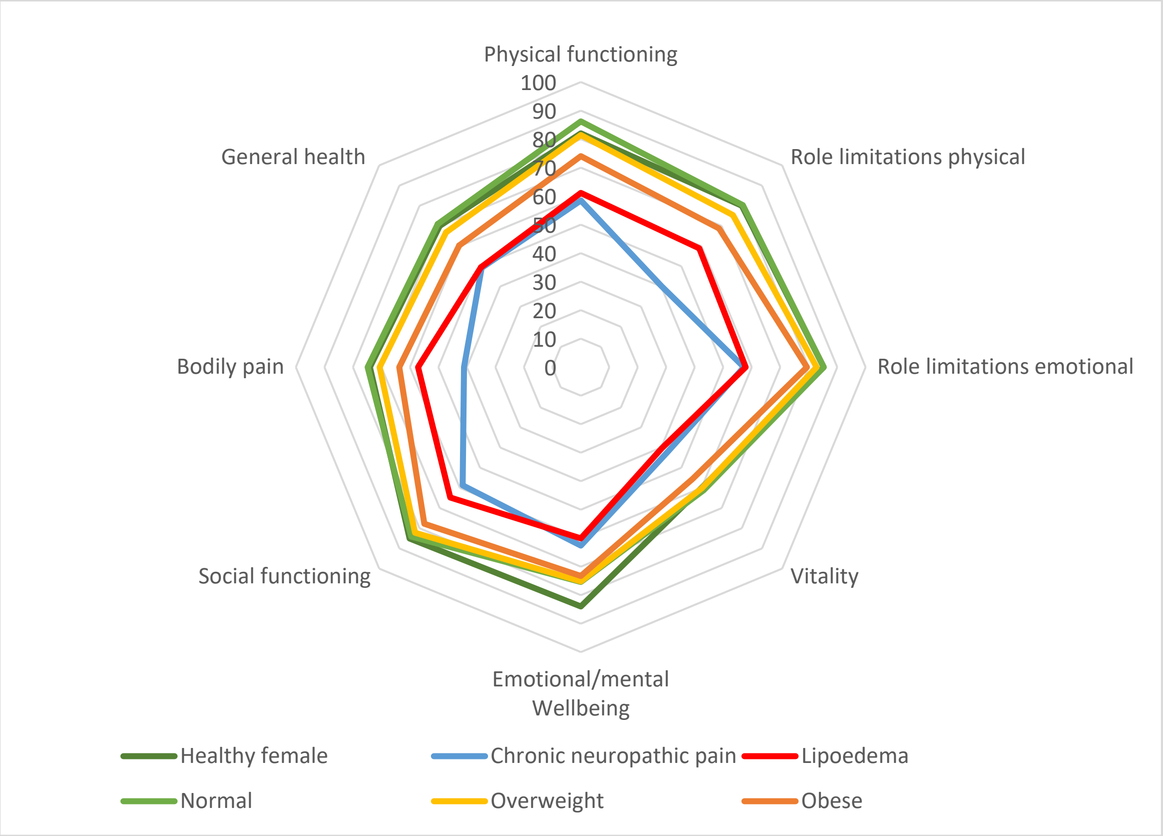

Supplement: S3 Fig — The lipoedema cases are comparatively similar to patients with chronic neuropathic pain (blue, SF36 data taken from Torrance et al. [3]). In contrast, overweight female without lipoedema (yellow, Sahle et al. [4]) are similar on many domains to healthy weight female (light green, Sahle et al. [4]; or dark green, Bowling et al. [5]), whilst obese female without lipoedema (orange, Sahle et al. [4]) show a lower score on some domains but not as low as the lipoedema cases. The healthy females from Bowling et al. (dark green; [5]) are age matched to the lipoedema cases, whereas the data from Sahle et al. [4] include males. The data on patients with chronic pain were taken from a general population of over 18 years old attending their GP service [3]. (PDF) [file pone.0274867.s012.pdf]
