# Supplementary material for: Investigation of clinical characteristics and genome associations in the ‘UK Lipoedema’ cohort
Source: PLoS One. 2022 Oct 13;17(10):e0274867. doi: 10.1371/journal.pone.0274867 (PMC9560129; doi:10.1371/journal.pone.0274867)
Supplement: Attachment — Submitted filename: PONE-D-21-18905_-_Response to review.doc [file pone.0274867.s013.doc]

**Response to Review**

Journal requirements:

When submitting your revision, we need you to address these additional requirements.

1. Please ensure that your manuscript meets PLOS ONE's style requirements, including those for file naming. The PLOS ONE style templates can be found at

https://journals.plos.org/plosone/s/file?id=wjVg/PLOSOne_formatting_sample_main_body.pdf and

<https://journals.plos.org/plosone/s/file?id=ba62/PLOSOne_formatting_sample_title_authors_affiliations.pdf>.

**Answer:** Our manuscript is now formatted according to your style requirements.

2. Thank you for stating the following in the Acknowledgments Section of your manuscript:

“The 100,000 Genomes Project is funded by the National Institute for Health Research and NHS England. The Wellcome Trust, Cancer Research UK and the Medical Research Council have also funded research infrastructure.”

We note that you have provided funding information that is not currently declared in your Funding Statement. However, funding information should not appear in the Acknowledgments section or other areas of your manuscript. We will only publish funding information present in the Funding Statement section of the online submission form.

Please remove any funding-related text from the manuscript and let us know how you would like to update your Funding Statement. Currently, your Funding Statement reads as follows:

“This project was supported by Lipedema Foundation (https://www.lipedema.org/) LF#006 (KG and PO), the Wellcome Trust (https://wellcome.org/) WT107064 (DBS), and the MRC Metabolic Disease Unit, and The National Institute for Health Research (NIHR) Cambridge Biomedical Research Centre and NIHR Rare Disease Translational Research Collaboration (https://www.mrl.ims.cam.ac.uk/mrc-metabolic-diseases-unit/) MRC_MC_UU_12012.1 (DBS). The funders had no role in study design, data collection and analysis, decision to publish, or preparation of the manuscript.”

Please include your amended statements within your cover letter; we will change the online submission form on your behalf.

**Answer:** We apologise for the mix up of where acknowledgement of funding appears. The ‘100,000 Genomes Project’ and ‘Understanding Society’ are stipulating the use of these phrases in any paper using their data, however, if necessary we can split these phrases into funding related statements and acknowledgement.

Thus, we can update the **‘Funding Statement’** to include the funding streams of both the 100,000 Genomes Project and Understanding Society. Therefore, please use the following text:

“This project was supported by Lipedema Foundation (https://www.lipedema.org/) LF#006 (KG and PO), the Wellcome Trust (https://wellcome.org/) WT107064 (DBS), and the MRC Metabolic Disease Unit, and The National Institute for Health Research (NIHR) Cambridge Biomedical Research Centre and NIHR Rare Disease Translational Research Collaboration (https://www.mrl.ims.cam.ac.uk/mrc-metabolic-diseases-unit/) MRC_MC_UU_12012.1 (DBS). The ‘100,000 Genomes Project’ is funded by the National Institute for Health Research and NHS England. The Wellcome Trust, Cancer Research UK and the Medical Research Council have also funded the 100,000 Genomes Project research infrastructure. ‘Understanding Society’ is funded by the Economic and Social Research Council and various Government Departments. The funders had no role in study design, data collection and analysis, decision to publish, or preparation of the manuscript.”

For an updated **‘Acknowledgement’** in the manuscript we suggest the use of the following:

“The authors thank all participants for volunteering their time for this study. We would also like to thank ‘LipoedemaUK’ for facilitating recruitment through their members. This research was made possible through access to the data and findings generated by the 100,000 Genomes Project and Understanding Society. The 100,000 Genomes Project is managed by Genomics England Limited (a wholly owned company of the Department of Health and Social Care) and uses data provided by patients and collected by the National Health Service as part of their care and support. Understanding Society is under the scientific leadership by the Institute for Social and Economic Research, University of Essex, and survey delivery by NatCen Social Research and Kantar Public. The research data are distributed by the UK Data Service. We also extend our thanks to members of the St George’s University of London (SGUL) Lymphovascular Research Team for invaluable discussions and feedback on our work and to the following members of the Lipoedema consortium: Dr Yann Klimentidis, University of Arizona; Prof Natasha Harvey and Dr Hamish Scott, University of South Australia.”

3. In your Data Availability statement, you have not specified where the minimal data set underlying the results described in your manuscript can be found. PLOS defines a study's minimal data set as the underlying data used to reach the conclusions drawn in the manuscript and any additional data required to replicate the reported study findings in their entirety. All PLOS journals require that the minimal data set be made fully available. For more information about our data policy, please see http://journals.plos.org/plosone/s/data-availability.

Upon re-submitting your revised manuscript, please upload your study’s minimal underlying data set as either Supporting Information files or to a stable, public repository and include the relevant URLs, DOIs, or accession numbers within your revised cover letter. For a list of acceptable repositories, please see http://journals.plos.org/plosone/s/data-availability#loc-recommended-repositories. Any potentially identifying patient information must be fully anonymized.

Important: If there are ethical or legal restrictions to sharing your data publicly, please explain these restrictions in detail. Please see our guidelines for more information on what we consider unacceptable restrictions to publicly sharing data: http://journals.plos.org/plosone/s/data-availability#loc-unacceptable-data-access-restrictions. Note that it is not acceptable for the authors to be the sole named individuals responsible for ensuring data access.

We will update your Data Availability statement to reflect the information you provide in your cover letter.

**Answer:** We have submitted our discovery data to Figshare and the DOI number for the data is: 10.24376/rd.sgul.16803109. Once we have a DOI number for the published paper, we will add that to the Figshare record and publish it.

4. We note that you have stated that you will provide repository information for your data at acceptance. Should your manuscript be accepted for publication, we will hold it until you provide the relevant accession numbers or DOIs necessary to access your data. If you wish to make changes to your Data Availability statement, please describe these changes in your cover letter and we will update your Data Availability statement to reflect the information you provide

**Answer:** As per (3) we have submitted our discovery data to Figshare.

5. Please amend the manuscript submission data (via Edit Submission) to include author Lipoedema Consortium, Genomics England Research Consortium^4^

**Answer:**

***Genomics England Research Consortium*** contributors:

The list of authors covered by the GEL consortium are here: <https://www.genomicsengland.co.uk/wp-content/uploads/2021/05/Genomics-England-Research-Consortium-author-list-2.pdf>

For a full name list go here: <https://www.genomicsengland.co.uk/wp-content/uploads/2021/05/Genomics-England-Research-Consortium-author-full-names-list-3.pdf>

The lead author is Richard Scott and his email address is [richard.scott@genomicsengland.co.uk](mailto:richard.scott@genomicsengland.co.uk).

***Lipoedema Consortium*** consists of the following four members:

Y.C. Klimentidis, Department of Epidemiology and Biostatistics, University of Arizona, Tucson, AZ, 85724, USA.

H.S. Scott and N.L. Harvey, Molecular Pathology Research Laboratory, Centre for Cancer Biology, SA Pathology and University of South Australia, Adelaide, South Australia, 5000, Australia.

P. Ostergaard, Molecular and Clinical Sciences Institute, St. George's University of London, London, United Kingdom.

Consortium lead: N.L. Harvey <Natasha.Harvey@unisa.edu.au>

6. Please include your full ethics statement in the ‘Methods’ section of your manuscript file. In your statement, please include the full name of the IRB or ethics committee who approved or waived your study, as well as whether or not you obtained informed written or verbal consent. If consent was waived for your study, please include this information in your statement as well.

**Answer:** We have now included the full name of the ethics committee which approved our study (pg. 6, line 101). Written consent was obtained from all participants, this is stated in line 101 of pg. 6.

Reviewers' comments:

Review Comments to the Author

**Answer:** We thank all three reviewers for their feedback on the manuscript and the important points they have raised. Where possible we have made suggested changes to the manuscript as detailed below. For some of the points raised, the current data will not allow us to dive deeper into the suggested aspects. This is something that we would like to consider for future data collection.

Reviewer #1: The article provides insights into the genetics of Lipedema that will potentially aid in defining the pathogenesis of the disease.

Comments:

1. Have the authors any information on the stage of Lipedema patients? It would be very interesting to correlate the SNP data with the different stages if possible. The authors might find the article by Herbst et al. helpful for the lipedema diagnosis (<https://pubmed.ncbi.nlm.nih.gov/34049453/>)

**Answer:** The staging system of lipoedema that the reviewer refers to had not been adopted in the clinics when many of the patients were recruited. Also, more than one classification system exists for lipoedema, but they are not universally used in the UK as we found they are of limited clinical use.

We did consider staging the patients and looked at various models, but as many of the patients were seen at various time points after their initial diagnosis it was not possible to stage them with any certainty for the study. Many of our patients would have been stage I or II on first clinical visit, but on recruitment to the GWAS study they might have progressed into stage III.

The study is also intentionally biased towards stage I and II as any recruits new to our clinics would only be included if BMI <40 and WHR <0.85. We therefore decided against using staging because what was required for phenotyping was a clear and unequivocal diagnosis not a severity score.

2. Have the authors considered analyzing the data between upper and lower limb data separately?

**Answer:** Unfortunately this data was not collected and therefore such an analysis is not possible. We will consider collecting this information going forward to facilitate future analyses.

3. The authors mentioned that SNPs might be associated with the biosynthesis of the hormones, Do the authors have any information on the hormonal status of the patients and/or the controls?

**Answer:** Hormonal profiles were studied previously in a cohort of lipoedema patients, but no abnormalities were ever found (pers. comm., Peter Mortimer). This situation is borne out by the literature where no hormonal abnormalities are proven. It is likely that lipoedema is hormone dependent, but more work is needed to elucidate the nature of this association and the underlying mechanisms.

4. The inclusion criteria included patients with BMI <40 with no abdominal Obesity. However, the range of BMI (19-58.8) is very wide. Have the authors considered analyzing the data based on the BMI classes (overweight vs obese)?

**Answer:** We thank the reviewers for their suggestion and agree this would be an informative analysis. Unfortunately BMI was not available for the control dataset and therefore a full stratified analysis was not possible. However we have now undertaken a case only analysis of BMI for the top GWAS SNPs to check if there is any relationship between BMI and genotype. No associations were identified perhaps supporting the assertion that weight gain is predominately related to disease progression rather than a distinct aetiology. We have updated the manuscript to read “A case only analysis of the discovery cohort identified no relationship between BMI and genotype of these top SNPs (P<0.01)“ on page 21 line 347.

5. The authors mentioned that LHFPL6 as a potential marker for Lipedema. Since not all lipedema patients have lipomas, have the authors considered analyzed the data by grouping non-lipoma vs lipoma lipedema patients?

**Answer:** Clinically lipomata may be present but not palpable particularly if an element of obesity is present and therefore not consistently recorded. We have seen lipomata or lipomata-like structures on US imaging in a few of our cases. In some individuals the fat deposits did not have the clear capsule usually associated with lipomata (unpublished) so further study is needed to elucidate this.

Others have reported that some patients have grainy, nodular fat (like “beans in a bag”) (Buck et al 2016; PMID: 27757353), and the question is, how this relates to lipomata? It is therefore possible that all lipoedema patients have lipomas but that they are not always detectable. Thus, the diagnosis of lipomas is not straightforward.

For these reasons we were not consistently collecting data on lipomata and are therefore unable to perform the suggested analysis. However, given our findings, we will seek to collect this information during recruitment going forward.

Reviewer #2: In their Study, Grigoriadis et al perform the first genome association study on lipedema patients, aiming to unravel the genetic background of the disease and allocate potential genes to lipedema that is still lacking biomarkers whereas the molecular background remains elusive.

While of great interest, the study requires extensive clarifications and further analysis of the already acquired data to strengthen the conclusions and outcomes of the analysis. A detailed list of the questions to be answered is below:

1. - What kind of physician set the diagnosis? Has an angiologist been involved to rule out presence of lymphedema or impaired lymphatic function? If yes, have any specific tests been undertaken for this purpose?

**Answer:** Angiology does not exist as a speciality in the UK. The nearest specialty is vascular surgery, but vascular surgeons in the UK do not specialise in lymphatic disease and would refer to a specialist department in diseases of the lymphatic system such as Derby, or St George’s in London, both of which are considered national centres in the UK for lymphoedema and lipoedema as recognised by the patients’ organisations: the Lymphoedema Support Network and Lipoedema UK and the British Association of Dermatologists.

All patients were examined for clinical signs of lymphoedema and venous disease. Lymphoscintigraphy was performed in a small number of lipoedema patients (e.g. to exclude primary lymphoedema presenting with a “fatty phenotype”, or to confirm the presence of early secondary lymphoedema). However, the majority of patients did not require lymphoscintigraphy as lipoedema remains a clinical diagnosis, and there were no clinical concerns that warranted further investigation.

2. - Please present the stage of lipoedema for the patients included in the analysis - this is not mentioned in the suppl. Material

**Answer:** As stated in response to reviewer 1, the authors carefully considered staging the patients but decided against this. Many of our patients would have been stage I or II on first clinical visit, but on recruitment to the GWAS study they might have progressed into stage III. We therefore decided against using staging because what was required for phenotyping was a clear and unequivocal diagnosis not a severity score.

3. - In Table 2 it is indicated that 39.1% of patients included are severely obese –

has a clustering based on BMI taken place? This would be advisable, even if the BMI of the control databases is not clear.

What is more, patients with oedema of the feet should be either excluded or analysed separately (probably lymphedema is present).

**Answer:** Obesity and lymphoedema are common associated conditions with lipoedema. One can still have a classic case of lipoedema based on history and physical characteristics yet, if longstanding, lymphoedema or obesity or both may have developed. It was a clear and unequivocal diagnosis of lipoedema that was considered most important for recruitment to this GWAS study, but as explained in the paper, any lipoedema cases with obesity were only included if they at earlier presentations to the clinicians had not shown obesity.

Whilst three patients with oedema of the feet are included in our study, we would reiterate that these cases have only been included if clear evidence of lipoedema is also present and based on their clinical history we were convinced they were clearly a lipoedema patient.

As mentioned under Q4 for Reviewer 1, we have conducted a case only analysis to look for a relationship between the reported top SNP genotypes and BMI but identified no associations. This has been included in the manuscript on page 21 line 347.

4. - In regard to bruising: commonly the patient reported bruising deviates from the clinically observed at the time of the clinical examination. What was the % of bruising present during the clinical assessment?

**Answer:** In our view, while accepting that bruising is a feature of lipoedema it is frequently seen in gynoid obesity and therefore is not an important discriminating criterion for diagnosis of lipoedema. For this reason, we did not discriminate between bruising observed during clinical assessment or from self-reporting in our database, but instead simply a recorded a yes/no status. It is therefore not possible to give a % of bruising present during clinical assessment.

5. - Has analysis of specific clusters taken place? In particular analysis of SNPs of specific lipoedema stage. This is essential to understand the dynamics of the disease, particularly given Stage IV (lipolymphoedema) is very much clinically different that Stage I-III lipoedema

**Answer:** There is a lot of debate about what lipolymphoedema is. Most patients with lipoedema and significant oedema have associated obesity. However, a small number without obesity have localised ankle oedema with no foot swelling. None of the cases included in the study would fall within the Stage IV (lipolymphoedema). With relation to staging of patients, as previously discussed, such information was not collected and therefore such an analysis is not possible.

6. - Previous articles published by other groups have already suggested potential gene involvement. These articles are neither cited nor discussed. Please include in your discussion. Articles: Felmerer et al J Surg Res (DOI: 10.1016/j.jss.2020.03.055) and Felmerer et al Sci Rep (DOI: 10.1038/s41598-020-67987-3)

**Answer:** We thank the reviewer for their suggestion. Felmerer et al (2020 in Sci. Rep) investigated if there is a link between lymphoedema and lipoedema and explored the blood serum levels of a list of ‘lymphoedema’ genes/proteins such as PDPN, PROX1, LYVE1, CCL21, VEGFR3, and VEGFC. These genes did not appear near the loci of our top SNPs in the GWAS (P<0.01). See Q7 for further details of our response to this topic.

Using similar methods, Felmerer et al 2020 in JSR identified the expression profile in their lipoedema cases and found CCND1 to be significantly upregulated and CEBPD, CFD, NCOR2, KLF4 to be downregulated. Although these gene candidates may play a role in the onset and development of lipoedema, they did not appear near the loci of our top SNPs (P <0.01).

Interestingly, the CEBPD gene reported by Felmerer et al 2020 in JSR gives a link to potential involvement of oestrogen in the course of lipoedema development and would fit with the association to the CPE gene we showed in our enrichment analysis and CYP3A4 and CYP3A5 in our pathway analysis. We have included a link to the Felmerer et al 2020 JSR paper in our discussion (see page 23, line 409).

7. - Whether lymphatic vascular involvement is a cause or results of lipoedema is still quite debatable. The authors are requested to discuss this aspect based on the SNPs they have identified

**Answer:** In our study we checked if any of the top SNPs are associated with the genes related to lymphatic development and, as the study did not highlight any, the topic was not discussed. This would follow the findings presented in the Felmerer et al 2020 Sci Rep paper who concluded that no lymphatic phenotype underlies lipoedema. We have added a reference to this point in the introduction (see page 5, line 62) and the discussion (see page 24, line 413).

Reviewer #3: This prospective evaluation is a beautifully executed and rigorously undertaken investigation, in an attempt to understand the genetic substrate of the puzzling clinical phenotype of lipedema. The authors are to be commended for the design and execution of their project.

As they acknowledge, a limitation of the investigation is the relatively small sample size for analysis. I feel that the discussion surrounding the project would be strengthened with two additional elements:

1. Traditional attempts to describe and understand lipedema divide clinicians into three broad camps: those that believe it to be a fat disorder, those that view it as a loose connective tissue disease, and those that ascribe the condition to an underlying microlymphatic disorder, for which there is some scant imaging and molecular support. It would be desirable to address the potential interrelatedness of these hypotheses, particularly in light of the GWAS findings of the study.

**Answer:** This is a good idea, so we have included a description of the different hypotheses in the beginning of the discussion (page 21, line 357 and 364). Although our findings might point in one direction, we did not want to go too deep into the discussion about this as we do realise the limitations of our study. We agree this would be an interesting topic for discussion, but this would be for a future review paper once we have more GWAS cohorts.

2. In addition, for the purpose of rigor, the investigators have excluded the subgroup of lipedema patients who present later in life. It would strengthen the manuscript to underscore the potential heterogeneous nature of the patient population. Were the latter group to have been analyzed, it is theoretically feasible to imagine that a different set of SNPs might have been uncovered, suggesting that the condition known as lipedema might actually be a heterogeneous grouping of presentations with some biological mimickry.

**Answer:** This is a very good point indeed and we have added this to the discussion (page 25, line 445).
