# Supplementary material for: Investigation of clinical characteristics and genome associations in the ‘UK Lipoedema’ cohort
Source: PLoS One. 2022 Oct 13;17(10):e0274867. doi: 10.1371/journal.pone.0274867 (PMC9560129; doi:10.1371/journal.pone.0274867)
Supplement: Attachment — Submitted filename: PONE-D-21-18905R2_Response to Review.docx [file pone.0274867.s014.docx]

PONE-D-21-18905R2

Investigation of clinical characteristics and genome associations in the 'UK Lipoedema' cohort

PLOS ONE

**General address from authors:**

We thank Reviewers 4, 5 and 6 for their time taken to review our manuscript. We have addressed all points raised in the following pages. Please notice that all line references refer to the ‘Manuscript w. tracked changes’.

We feel the valuable points raised by the Reviewers and which has led to various changes, have improved the manuscript. We hope that our response satisfies the Reviewers and that they agree to these revisions.

**Reviewer #4:** Its very interesting study which involve more than 130 patients genetic data. Study developed some new knowledge about disease which will be helpful for future disease description.

Answer: We are very grateful to this reviewer for their kind comments.

**Reviewer #5**:

1. Is there an estimate of the prevalence of lipoedemia in the population? This would be useful to be added to the introduction. If there is no estimate then that should be discussed? Do the authors have an idea of what the incidence is based on their recruitment of cases? Or if the incidence is rising in line with rising rates of obesity?

Answer: There is a paucity of high-quality prevalence studies on lipoedema in the literature. Two groups in Germany have published an estimated prevalence of 10-11% in the overall female population, but this seems a little high in our opinion (Foldi et al 2006, Marshall et al 2011). Based on our own regional dermatology department we have previously estimated a prevalence of 1 in 72,000 but is likely to be an underestimate due to misdiagnosis in the community (Child et al 2010).

Clearly further work is needed to determine the real prevalence data. One obstacle to this is the current lack of a test to confirm the diagnosis, but hopefully this will improve in the future. Clear prevalence data would then allow us to confirm some researcher’s suspicions that incidence may be rising in line with rising obesity rates.

We have added some information related to this in lines 58-61.

2. This is just a comment, but lipedema appears to be quite rare and I wonder if a GWAS approach is appropriate for this disease. It is hypothesized that autosomal dominant variants are likely to cause lipoedemia (i.e. rare frequency variants, PMID: 20358611), so would suggest sequencing studies of well-phenotyped, familial samples would be a suitable approach to identify genetic risk factors. The authors mentioned that no monogenic cause has been identified for lipedema but I’m unsure if this is because these sequencing studies have not been performed?

Answer: Since submission of this manuscript, one paper has been published (PMID: 32872468) on a possible genetic cause of familial lipoedema. However, the study only includes 1 family and there is no functional validation of the variant, so we are still to see if this is real. Therefore, the genetics underlying lipoedema is still uncertain.

Whilst many patients present with a family history compatible with Mendelian inheritance (PMID: 20358611) other patients report no family history. By undertaking studies such as this we aim to explore the underlying architecture of the genetics of this disease. We are concurrently embarking on a research program using exome and whole genome sequencing to identify rare highly penetrant mutations in cases with a strong family history to further elucidate the genetics of this disease and we can reveal to the reviewer that we are seeing a large genetic heterogeneity in the familial cohort.

We have updated the manuscript to include the findings in PMID: 32872468, see line 87-88.

Most of my comments relate to the GWAS methodology and result reporting.

3. Were duplicate samples assessed for genotyping (not duplicates between the two batches, but duplicates assessed in the same batch)? This should be performed as standard for genotyping studies and SNPs which are not concordant (e.g. concordance <98%) between duplicates removed.

Answer: Duplicates within batches were not performed in the study, however, poorly performing SNPs would have been removed by our stringent QC including call rate and HWE analysis. Furthermore, additional poorly performing SNPs were identified and removed during the comparison between batches where 4 SNPs were identified and excluded. We have made it clearer in the manuscript that duplicate samples between batches were assessed and SNPs showing inconsistencies removed. (See line 130-131).

4. As the study includes only female participants, was X-chromosome heterozygosity assessed to investigate whether there were any suspected male genotypes, XO or XXY individuals?

Answer: We did indeed confirm genetically that all participants were female by looking at the inbreeding coefficient of the X-chromosome. We apologise for the omission from the methods section and have edited the manuscript to now state “All samples were confirmed as female using the PLINK sexcheck function (F inbreeding coefficient, <0.2 for females).” (See lines 154-155).

5. Was there a reason imputation was not performed for the study?

Answer: We did not perform imputation in this study due to the cases and controls being genotyped on the same SNP chip and potential uncertainty due to the low case sample size.

6. The authors have used a MAF>0.01 as a quality control cut-off. However, given the very small case numbers included in this study, I strongly recommend a MAF>0.05 be used. Very large odds ratios, particular those >3, are not compatible with a common SNP-disease model. The variants which have large ORs in this study (2 SNPs OR>3, 1 SNP OR>2.5) all have MAF<0.05 in controls, and I would consider the estimates for these variants to be unreliable.

Answer: Whilst we acknowledge the estimates of rare alleles can be inaccurate in small datasets, we would still like to include these results in our publication but have added the comment - “Given the modest sample size of this study, the confidence intervals of rare allele frequencies (MAF < 0.05) are likely to be wide.” (See lines 334-335 and Table 4). This should highlight to the reader that caution should be applied to these results.

7. What does “suggestive” mean in terms of suggestive genomic loci? What is the cut-off for calling something suggestive?

Answer: We use the term “suggestive” where we identify a variant with *p* < 1.0x10-4 in our discovery cohort and same direction of effect in the replication cohort. We have made this clearer in the paper (see lines 327).

8. When assessing whether a SNP affects expression of a gene, the eQTL signal should be assessed for colocalization with the phenotype signal. The latest GTEx analysis found that >90% of SNPs were associated with expression of at least one gene, in at least one tissue using a nominal P-value <0.05. Doing a simple look-up and using an arbitrary P-value cut-off for eQTL assessment is not good practice.

Answer: We have now performed colocalization analysis of the top eQTLs using the software LocusFocus which implements the frequentists colocalization method. As expected not all signals were found to colocalize and the manuscript has been updated to reflect this (see lines 32-36; lines 175-179; lines 362-363; lines 367-373; inserted new table – see the S8 Table; inserted new figure in supplementary - see S3 Fig.)

9. Have the identified loci in this study been identified as associated with other related traits by GWAS (e.g. waist-hip ratio, body proportions – e.g. leg/arm/trunk fat ratio, steroid hormone levels etc)?

Answer: None of the identified SNPs have been previously associated with related traits as documented by the GWAS catalog.

10. The study cohort is way too small to perform case-only analysis of the relationship between BMI and genotype. I recommend this is removed and mentioned only as a limitation of the study that this type of analysis could not be performed.

Answer: We agree that this type of analysis is not well powered with our sample size, however the analysis was requested by a previous reviewer, so we will keep the analysis in the 2^nd^ submission of the paper but with a revised statement now reading: “Although likely underpowered by our modest sample size, a case only analysis of the discovery cohort identified no relationship between BMI and genotype of these top SNPs (P<0.01).” (See lines 365).

11. “Approximately half the recruited women reported a family history of large legs, and this is consistent with the estimated SNP-based heritability of 50-60% calculated in the discovery cohort, indicating a strong genetic link to lipoedma.” This sentence is incorrect and should be deleted. Are the authors suggesting that the entire familial heritability of lipoedema to due to common genetic variation? I would suggest this is not the case, as with most complex diseases, heritability would be due to a number of factors including low-frequency, high-risk variants and environmental risk factors. Importantly, the SNP heritability is unreliable with very large standard errors – SEs are, in fact, larger than the SNP heritability estimates. Related to this, the authors have used prevalence estimates of 5% and 10% in their SNP heritability estimates but have not provided justification for this.

Answer: We thank the reviewer for pointing this out and have edited the sentence in the paper as follows:

“We estimated SNP-based heritability of 50-60% in the discovery cohort, indicating a strong genetic link to lipoedema. However, larger lipoedema cohorts are needed to validate this estimation. Strong association of autosomal dominant inheritance with sex limitation has been observed within affected family members with lipoedema” (See lines 387-388).

12. The authors mention that “fine-mapping analysis results showed…” however, there was no indication in the manuscript that fine-mapping was performed? What do the authors mean by fine-mapping?

Answer: We thank the reviewer for spotting this mistake and have edited the text to now read “…eQTL analysis results showed...” (See line 394).

13. The authors also state “finding from our enrichment analysis”, but then proceed to discuss eQTL look-up results. This is not an enrichment analysis.

Answer: As the signals discussed in this paragraph were not identified as colocalising signals we have adopted a cautious approach and removed these sentences from the discussion and abstract. (See lines 367-373, 419-429 and 32-36).

14. Given the relationship between obesity and lipedema, it could be useful to construct polygenic scores for obesity measures in your cohort and assess whether these are significantly different between cases and controls.

Answer: We investigated the possibility of applying a polygenic risk score for obesity (taken from the PGS catalog) to our data. However, whilst we acknowledge it would be of likely interest to the reader, given available polygenic scores for obesity are likely to include a substantial number of lipoedema cases, we anticipate the results may not be straightforward to interpret. We also think this work is outside of the scope of this project, but we will certainly look at exploring this in future projects when additional lipoedema cohorts exist.

15. Can lipedema cases be identified from the UK Biobank cohort? These could be used as another independent replication set, even if not as well-phenotyped as your discovery set.

Answer: We agree this is an interesting idea and a GWAS analysis of a “lipoedema” type using UKBB data is currently underway by an external colleague in a separate study.

16. I highly recommend that summary statistics for GWAS is submitted to the GWAS Catalog to be accessed by the scientific community.

Answer: We agree that submission to the GWAS Catalog would be useful for the community. In order to link with the publication, we will submit the data once the publication has been accepted and has a Pubmed ID. We will liaise with the Editor to ensure our data availability statement gets updated to reflect this.

**Reviewer #6:** The paper is one of the first to describe a GWAS analysis on a very important health issue affecting predominantly women, lipodema. It well written and the bioinformatics relating to the study design is robust. To that end, I have some questions:

1. The authors describe a ‘strict’ selection criteria on how the patients were recruited. As they are aware, the cohort numbers and choice is critical to a meaningful outcome. What precautions were taken to exclude patients with any underlying health conditions such as thyroid problems, vascular defects, cardiovascular, cancer, diabetes. There is a very large variation with BMI- how did the authors select the non lipodema cohort? What criteria was applied here- were they BMI matched age matched controls? If so, then are they comparing lipodema to obesity? With regards to the upper body, what criteria was used to ascertain minimal fat- callipers and fold test? What would the cut off be for this if minimal upper body obesity was used as a selection criteria?

Answer: Lipoedema does not appear to be linked with thyroid problems, vascular defects, cardiovascular, cancer, diabetes. Our previous studies (e.g. Child et al 2010; PMID: 20358611) included full work up for thyroid, diabetes, hormonal profile and cardiovascular abnormalities. None were identified. Therefore, the authors did not feel it appropriate to repeat the workup with this cohort of patients (as it would be a waste of precious resources). The authors took a detailed medical history, clinical examination and had access to the GP records which excluded any of the aforementioned issues.

Some of the most important aspects when selecting the non-lipoedema cohort to use as controls in the study was to find a large enough cohort of females of similar ancestry to our cases and ideally genotyped using the same SNP chip. The cohort from the Understanding Society: UK Household Longitudinal Study (<https://www.understandingsociety.ac.uk/about/who-are-our-participants>) fulfilled those criteria. It was not possible to do a BMI-match, or age-match as we did not have that information.

With regards to upper body fat, we relied fully on clinical examination. Rather than relying solely on BMI, we found the waist-hip ratio (WHR) a good way of differentiating from/excluding any obese patients, and thus for most of our cases, waist and hip circumference was measured. As described in the supplementary, patients whose WHR was above 0.85 at the time of the recruitment consultation were included in the study, but only if they had been known to the senior clinician for several years and historically would have fallen within the inclusion criteria. Any patients new to our service with a WHR >0.85 were not included in the analyses. However, it is a really challenging area which hopefully will be addressed in future studies.

2. How were the patients diagnosed with lymphoedema? Were the patients lymphatics measured by lymphosintigraphy? Did the authors identify any SNPs in the patient with venous issues such as Sox18? Do the authors believe that lymphoedema is a secondary side effect of excessive fat formation rather than the cause or an initiator of lipodema? There was a significant degree of oedema and bruising in patients- did the authors identify any SNPs in VegfA related pathways?

Answer: (a) Regarding how the lymphoedema was identified in the patients and if the diagnosis was measured by lymphoscintigraphy. The lymphoedema was diagnosed based on expert clinical examination (>20 years’ experience in a national lymphoedema clinic). The paper states that we elected not to investigate the intermittent mild oedema further as it was deemed secondary to vein issues. No lymphoscintigraphy was used in this study.

(b) No SNPs in or near *SOX18* were identified in any of the patients with vein issues.

(c) Regarding whether lipoedema causes lymphoedema or vice versa is a very good question. No convincing scientific information is available to answer this yet. The authors suspect it may be a side effect of excess adipose tissue rather than the initiator of the lipoedema, but cannot prove it.

(d) We did not identify any SNPs in or near *VEGFA* in any of the patients included in the study.

3. The SNP identified as key in lipoma is interesting. How many of the cohort patients were diagnosed with lipoma or dercums disease? Was this used in the selection criteria as a possible way to segment the cohort?

Answer: None of our recruits were diagnosed with Dercum’s. In this cohort only one patient had a well circumscribed lipoma. However, it was not something we routinely questioned the patients about. Future studies will involve closer interrogation and inspection for lipomas in case they are more prevalent than we originally thought.

4. Have any of the SNPs been validated? I would like to see a few of these validated by Sanger to strengthen the paper. This would be a simple experiment.

Answer: We were able to identify 19 samples from our dataset that had also been whole genome sequenced as part of the 100k Genomes Project. Comparing the genotypes of these 19 samples across the 9 replicated SNPs we find 100% concordance in genotyping.

We have amended the text to read: “Nineteen samples with SNP chip genotypes were also available as whole genome sequencing data from the GEL Project. Comparison of selected SNPs (n=9, top SNPs from replication) identified a concordancy of 100% between platforms.” (See lines 147-149).

5. Can the authors speculate about the size of the cohort that would be needed to provide a statistical confidence in the analysis.

Answer: Just based on what p-values we would get if case/control ratios were the same in the replication as in the study, then a replication cohort of 100 cases and 200 controls should be sufficient to replicate the top loci (OR=2, p<0.005, replication of 10 SNPs with Bonferroni type correction). The Uni-Michigan power calculator suggests we need 200 cases and 400 controls for ~90% power to detect a variant at 0.005 (OR=2, prevalence=0.1). We have just been awarded additional funds to continue collecting samples and hope to be able to perform such a replication within the next two years.
