# Supplementary material for: Investigation of clinical characteristics and genome associations in the ‘UK Lipoedema’ cohort
Source: PLoS One. 2022 Oct 13;17(10):e0274867. doi: 10.1371/journal.pone.0274867 (PMC9560129; doi:10.1371/journal.pone.0274867)
Supplement: Attachment — Submitted filename: PONE-D-21-18905R3_-_Response to Reviewers.docx [file pone.0274867.s015.docx]

PONE-D-21-18905R2

Investigation of clinical characteristics and genome associations in the 'UK Lipoedema' cohort

**Comments to the Author**

**Reviewer #5: The authors have done a good job in addressing my previous comments. I just have a few further queries.**

**Q1. I assume the authors have a typo answering my question regarding imputation – that the cases and controls were NOT genotyped on the same platform. This is a hurdle, but could be easily rectified by harmonizing the two groups to the same SNP lists before imputation. I don’t see that the smaller case size would have an impact on the reliability of the imputation. I don’t expect the authors to impute for this study, but would recommend for future work.**

Answer: We thank the reviewer for their comments and will certainly implement imputation as a standard for any future studies. The reviewer is indeed correct that our cases and controls were not typed on the same chips, but rather on broadly overlapping Illumina chips, we apologise for this error in our explanation.

**Q2. I still strongly recommend that SNPs that a MAF > 5% is used for the study. For a sample of 148 cases, only 7-8 cases need to be carriers for testing. The low allele frequencies, high ORs and wide confidence intervals suggest these results to be likely driven by statistical artefact. As previous, the ORs for these variants are too high to be compatible with a common variant model. I also note that the table doesn’t provide CIs and a table footnote is not enough to warn readers for interpretation.**

Answer: As suggested, we have removed the three SNPs with a MAF<5% from the results including Table 4, S5 Table, Fig 2 and have updated the methods (see line 159) and results section (see lines 330-332) accordingly. Counts of SNPs taken forward to replication (with MAF>%5) have been edited in the document where appropriate (e.g. lines 146, 165-166 etc).

**Q3A. I appreciate that the authors have performed colocalization analyses. There should be futher information provided, including which GTEx dataset was used (v8 or v7?).**

Answer: The GTEx database used in the analysis was v7. This has now been included in our methods section (see line 172).

**Q3B. Given the large number of genes and tissues assessed, the multiple testing burden would have been high. Could the authors provide what their cut-offs were in light of this (for eQTL p-value and colocalization SSP)?**

Answer: In the legend for S7 Table we report “significant” **eQTL** results as those with P<5x10^-6^, reflecting a Bonferroni correction relaxed to adjust for the highly correlated nature of the tests. We acknowledge that this is not a rigorous solution to the multiple testing problem. Rather than perform a permutation-based correction at this stage and given the new focus on the more informative colocalization analysis, we now only report eQTLs as significant with P<6x10^-8^ (strict Bonferroni correction based on total number of tests) whilst retaining all results (P<0.001) in S7 Table for reference. We have updated the methods (lines 172-175), results (lines 346-349 and 359-364), discussion (lines 393-395) and Supplementary Tables legend sections accordingly.

With regards to multiple testing in the **colocalization** analysis, we report in S8 Table all results (simple sum P-value>0) and highlight as significant those with SSP>3 (as recommended in the LocusFocus publication). In our case this corresponds to the Bonferroni corrected *P*-value (17 tests of colocalization, P<0.01, SSP=3.2). We have now clarified this in the methods (lines 179-181) and results (lines 365 – 367), edited the SSP threshold to be 3.2 (lines 181 and 367) and edited S8 Table legend.

We had performed our GO enrichment analysis with all genes tested for colocalization rather than those that were statistically significant. Given only two genes pass the threshold of significance, we have now removed the GO enrichment analysis from our methods (deleted line 183), results (deleted lines 370-374), discussion (deleted lines 385-386) and deleted the S9 and S10 Tables and legends.

**Q3C. It looked like there was evidence of colocalization for rs10499948 and LHFP in pancreas (ST8) but this wasn’t in fig S7, why was this? Also there was some evidence for LHFP in liver in fig S7 but this wasn’t in ST8. Why?**

We believe the reviewer is referring to rs1409440 in their comment and not rs10499948, and S3 Fig not S7 Fig. There was an issue with the formatting of S3 Fig (downloaded from LocusFocus) where alternate y-axis tissue labels were missing, which is why there are apparent differences between S3 Fig and S8 Table; i.e. the yellow (now red) box in S2 Fig refers to evidence of colocalization in the pancreas not liver as labelled. We have created our own custom output plot from the data now to illustrate the correct results for this locus and this replaces the original S3 Fig as (A) in S2 Fig. We have also produced the same figure for the other significant colocalised gene signal, which now is presented as (B) in S2 Fig.

**Reviewer #6: The authors should validate the a selection of SNPs identified to ensure the conclusion is sound. It is not clear that the normal cohort is classified as obese or not. This could potentially impact the findings.**

Answer: We thank the reviewer for their comment, however, after discussion with the PLoS ONE editorial team, we do not feel that this is essential given the technologies used in this study. The findings reported in this paper are suggestive only, and hopefully in the future additional replication series will be identified to validate the specific associations.
